# The impact of affective states and traits on perceptual stability during binocular rivalry

**DOI:** 10.1038/s41598-023-35089-5

**Published:** 2023-05-17

**Authors:** Nils Kraus, G. Hesselmann

**Affiliations:** 1 Psychologische Hochschule Berlin (PHB), 10179 Berlin, Germany; 2 Freie Universität Berlin, 14195 Berlin, Germany

**Keywords:** Psychology, Perception, Emotion

## Abstract

Affective states and traits have been associated with different measures of perceptual stability during binocular rivalry. Diverging approaches to measuring perceptual stability as well as to examination of the role of affective variables have contributed to an inconclusive pattern of findings. Here, we studied the influence of affective traits, such as depressiveness and trait anxiety, and states, which were manipulated with a musical mood induction paradigm, on different measures of perceptual stability (dominance ratios and phase durations) during binocular rivalry. Fifty healthy participants reported alternations in two conditions: a biased perception condition with an unequal probability of perceiving stimuli, using an upright versus a tilted face with a neutral expression, and a control condition with equal chances of perceiving stimuli, using Gabors of different orientations. Baseline positive state affect significantly predicted longer phase durations whereas affective traits did not yield any such effect. Furthermore, in an exploratory analysis, induced negative affect attenuated stimulus related bias in predominance ratios. Overall, we found a strong correlation between both measures of perceptual stability (phase durations and dominance ratios). Our findings thus question the distinction between different measures of perceptual stability during binocular rivalry and highlight the role of affective states in its formation.

## Introduction

When the brain is confronted with ambiguous sensory inputs, what typically happens is a back and forth switching between the plausible alternatives within perceptual experience (e.g., when viewing the Necker cube). This phenomenon of multistable perception has been frequently used to study a potential link between perceptual experience and other mental variables that are typically considered to be independent of perception^1^. Findings suggest influences from a diversity of extraperceptual constructs such as creativity, conscientiousness or autistic thinking style on temporal dynamics during multistable perception^2–4^. All of these findings have been intensively discussed in the general debate over the cognitive penetrability of perception (CP^5^). Cognitive penetrability refers to the notion that higher-level cognitive processes or mental variables can directly influence the content of perceptual experience. Several researchers have questioned the possibility of extraperceptual variables ever being able to change what or how we see. Rather, they suggest, the described findings are best understood by extraperceptual variables influencing response behaviour or allocation of attention, but not perceptual experience per se^6^.

A major candidate in the race for mental processes that could penetrate visual perception has been emotional experience in the form of affective states and traits (i.e., transient affective sensations and long-lasting dispositional tendencies, respectively). Several authors have argued for the *affective realism* hypothesis, wherein the influence of emotional variables would not be limited to guiding attention allocation. Rather, affective realism proposes that affect continually shapes current perceptions, including those of affectively neutral stimuli^7^. Siegel et al.^8^ found that when participants were exposed to an experimentally masked scowl face, they rated a visible neutral face more negatively. The authors argued that this was not due to top-down factors like judgment but rather the integration of affective information into sensory input. Notably, the effect only occurred when both faces were presented simultaneously, not sequentially, suggesting it was not due to priming or misattribution^9^. Although several studies showed that stimulus valence influences the perceptual processing of it (i.e., the probability of the stimulus being perceived and not its competitor^10,11^) this is usually not considered as an example of CP, as it is not an observer based variable influencing perceptual experience, but a feature of the stimulus itself. However, these attentional biases have been shown to be amplified in affective and anxiety disorders^12,13^.

Notably, alternation rates (i.e., how often perception switches between both stimulus variants in a given time interval) during binocular rivalry have been shown to be significantly altered in those disorders not only with respect to aversive stimuli, but also in affectively neutral stimuli like gratings^14–16^. In a study by Jia et al.^15^, different types of affective traits correlated with alternation rates during binocular rivalry. The average phase duration (i.e., the time it takes for perception to switch to the previously neglected stimulus variant) for affectively neutral gratings was almost twice as long in depressed subjects as it was in highly anxious subjects. However, results of these studies have been partly conflicting. For example, alternation rates in depression have been attenuated in some studies^14,15^ but not in others^16,17^. Furthermore, it remains unclear whether the described effects are indeed attributable to altered emotional experience and not to some third variable that is also affected in patients. For example, processes like attention shifting and intentional control can crucially determine phase durations during rivalry^18^ and have been shown to be altered in affective and anxiety disorders^19^.

Until now, only limited research has been conducted on potential influences of affective states and traits on rivalry dynamics of affectively neutral stimuli in healthy participants. Sheppard and Pettigrew^20^ found a strong correlation (*r* = 0.8, *p* < 0.005) between positive state affect and dominance ratio (i.e., the proportion of total viewing time with which a particular stimulus variant was perceived) of the two competing percepts during plaid motion rivalry. However, this result is based on a small sample size of only ten participants. Nevertheless, similar results have been reported in another study^21^ in which positive mood state correlated positively with disappearance phases during motion induced blindness, a phenomenon that is typically considered as a form of bistable perception. In an experiment that was primarily aimed at studying how different stimulus features affect phase durations, Law et al. found a negative correlation of trait anxiety and depressiveness and average phase duration in a sample of healthy participants^22^. However, the correlations were small (*r*'s = 0.22–0.27) and were found to be significant only in some stimulus variants but not in others. The study also did not find any significant correlation between subjective mood state and phase durations or predominance ratio of both stimulus variants, challenging the aforementioned results. Overall, research on the relationship of affective state and traits and altered processing of affectively neutral stimuli during bistable perception has yielded conflicting results, making further research worthwhile.

Here, we wanted to investigate a potential relationship between affective states and traits with different measures of perceptual stability in healthy participants during binocular rivalry. Based on the findings of Jia et al.^15^ we hypothesized that phase durations of affectively neutral stimuli would correlate positively with depressiveness and negatively with trait anxiety. Furthermore, based on the studies of Pettigrew and Carter^21^ and Sheppard and Pettigrew^20^, we expected state affective valence to alter the predominance ratio of the competing stimuli during binocular rivalry. More specifically, since a previous study of ours^23^ indicated a potential link between positive affect and stronger reliance on priors in visual perception, we reasoned that positive affect would increase pre-existing biases and negative affect would reduce the same biases.

## Methods

All experimental procedures, sample size, research hypothesis, exclusion criteria as well as statistical analyses were preregistered prior to data collection and can be accessed via aspredicted.org/S9J_TR9. All collected data, visual stimuli as well as the analysis script are publicly available under https://osf.io/2gyvu/.

### Sample

The sample consisted of 59 participants who were recruited from a student pool as well as the general population and received either monetary compensation or course credit. We excluded 5 participants because they reported less than 15 reported alternations (i.e., button presses) in any of the experimental conditions. This is likely due to the fact that one stimulus variant was intended to lead to perceptual dominance over the other (upright face > tilted face, see section on visual stimuli), which resulted in reduced perceptual alternations in this condition. We further had to exclude two participants due to technical errors during the stimulus presentation and two because they reported mixed percepts on more than 20% of the time, resulting in the final preregistered sample size of 50 (33 female, 43 right-handed, age = 27.94, SD = 10.99). All participants had normal or corrected-to-normal visual acuity. Informed written consent was obtained from all participants after they received a detailed written description of the study. The experiment has been conducted according to the principles expressed in the Declaration of Helsinki and has been approved by the local ethics committee at Psychologische Hochschule Berlin (PHB).

### General procedure

Participants were seated in front of a 24″ LCD screen (Acer KG241, 75 Hz) in a dimly lit room. They were asked to place their head on a chinrest 60 cm in front of the screen, and view the stimuli through a stereoscope (Screenscope, LCD version). Questionnaires measuring affective traits depressiveness (PHQ-9^24^), anxiety (STAI-T^25^) as well as momentary positive and negative affect (PANAS^26^) were completed by the participants before they were asked to select a preferred musical stimulus, which is described in detail in the next section. After this, participants were familiarized with the general phenomenon of binocular rivalry and given instructions over the specifics of the task. During a practice run, participants were verbally instructed to report any change in their current perception by pressing a button (e.g., pressing the left arrow key if they see an upright face and pressing the right arrow key as soon as the tilted face becomes the dominant percept). They were also informed that they could report a mixed perception if neither stimulus variant appeared to reach clear dominance over the other (down arrow key). One experimental block consisted of eight trials, in each of which two competing stimuli were presented for 90 s. The three experimental blocks differed by the valence (positive, negative, neutral) of the mood induction, which consisted of 30 s of listening to an acoustic stimulus before every trial (see next paragraph). In total, the experiment lasted between 50 and 60 min.

### Auditory mood induction

**Figure 1:**
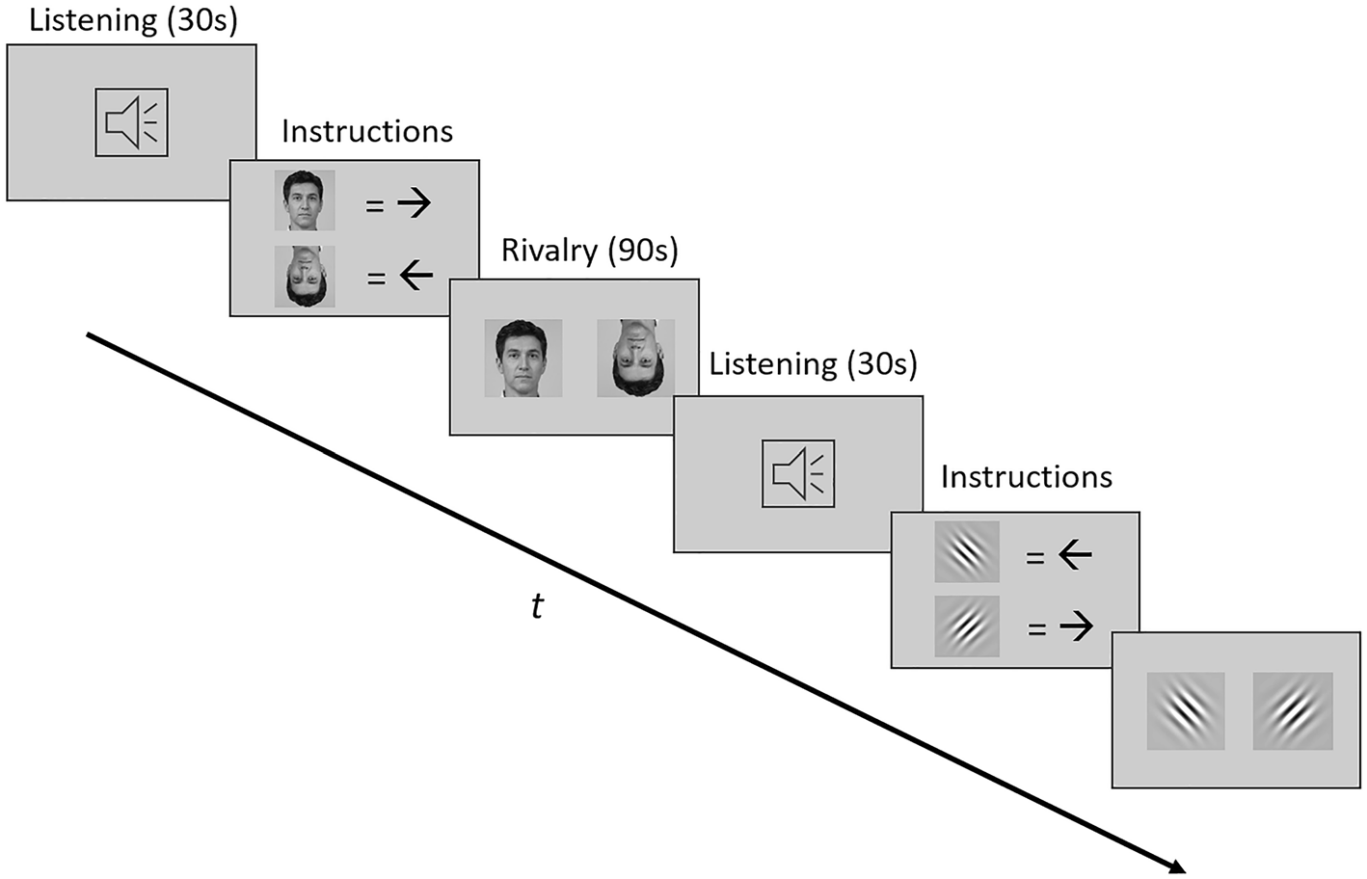
Experimental paradigm. The beginning of every trial consisted of 30 s in which participants either listened to a harmonic musical stimulus (positive valence condition), a scrambled musical stimulus (negative valence condition) or no auditory stimulus was presented (neutral condition). Subsequently, instructions on button assignment were presented (i.e., which button should be pressed when a particular stimulus variant is perceived during the trial). Afterwards, two competing stimulus variants were presented to the different eyes through a stereoscope, creating binocular rivalry. Stimuli were shown for 90 s, during which participants were asked to report changes of their current percept via button press.

The mood induction paradigm was adapted from a recent study of ours on the relationship between affect and visual perception^23^. The paradigm is based on the premise that the preference for a musical stimulus is determined by its predictability and information content^27–29^. To induce positive affect, participants were asked to choose their preferred piece of music from among five preselected harmonic stimuli. All stimuli were instrumental and polyphonic, with a tempo between 90 and 105 bpm. In the positive valence condition, the chosen piece of music was separated in to eight elements, each lasting 30 s, which were then played in a consecutive order to the participants before every binocular rivalry trial (see Fig. 1). To induce negative affect while holding the low-level features of the acoustic stimulus (such as volume, pitch, and tempo) constant, the predictability of the chosen piece of music was significantly reduced. This was achieved by separating the stimulus into short sound bites (ranging between 150 and 300 ms) and rearranging them in a random order. The resulting auditory stimulus was again separated in to eight different 30 s elements, one of which was played to the participants before a given trial in the negative valence condition. Kraus et al.^23^ established that listening to both stimulus variants (harmonic and scrambled) leads to significant changes in reported affective valence, with lower predictability leading to more reported negative affective valence. Since parallel auditory stimulation has been shown to influence phase durations during binocular rivalry^30^, the acoustic stimuli were played before and not during visual stimulus presentation. As every experimental block consisted of eight trials with 30 s of listening time, the overall duration of mood inducing stimuli amounted to four minutes in every block. Auditory stimuli were played on Sennheiser HD-25-1 II headphones. In the neutral condition, participants were simply asked to rest during the 30 s before presentation of the visual stimuli started and no auditory input was played. The order of stimulus valence conditions was counterbalanced across participants.

### Visual stimuli

Stimulus presentation and response detection were controlled using the PsychToolbox software package^31^ for Matlab (R2019b, MathWorks Inc., USA). Two sets of visual stimuli were presented to participants through a stereoscope at a size of 3.5° visual angle on a grey background. Stimulus sets were selected in a pilot experiment (n = 5). In this pilot study, we tested five different stimulus sets (letters, numbers, faces, spheres, Gabors) for how strong of a bias was created by presenting them in their standard alignment to one eye versus a tilted (180°) stimulus variant to the other eye. This procedure was chosen to either induce a consistent bias towards one stimulus variant over the other or as low of a bias as possible, while ensuring equal low-level visual features between stimulus variants. The first stimulus set consisted of diagonally oriented Gabor patches (− 45° and 45° from vertical; frequency = 6 cycles/°) which resulted in equal reported perception times of both stimulus variants during the pilot experiment. The second stimulus set consisted of an upright and a tilted face (180°), which in the pilot experiment led to the upright face being reported to be perceived about 60% of the time. To account for possible biases of eye dominance or preference towards a specific response button, side of stimulus placement on the screen as well as button assignment (i.e., assignment of perceived stimulus variant with the right or left arrow key) were randomized across trials within a block. Before every trial, participants received a visual description of which stimulus set they would see and the specific button assignment for that particular trial.

### Statistical analysis

In a first step, participants who reported mixed percepts on more than 20% of the time (n = 2) were removed from the dataset. For all further analysis, time periods in which subjects reported seeing mixed percept were excluded from the analysis. Furthermore, we excluded all responses that indicated a stable percept of over 30 s (0.47% of all responses), since a typical phase duration spans between 2 and 10 s and particularly long intervals could indicate low attention to the task^32^. Phase durations were thus calculated as the time between a button press that indicated the perception of one of the stimulus variants (left or right arrow) and the subsequent button press. To account for the commonly observed skewness of phase duration data and to investigate the influence of continuous questionnaire data, we preregistered the main analysis to consist of a generalized linear mixed effect model (GLMM) in which phase durations during neutral mood are predicted by stimulus set (faces, Gabors) and four questionnaire values (PHQ-9, STAI, PANAS scales positive and negative affect). Phase durations were assumed to follow a gamma distribution^33^. However, regression diagnostics indicated non-normality of residuals. We therefore compared model fits of four potential parametric distributions (normal, lognormal, Weibull, gamma; fitdistr package for R^34^) to the observed phase duration values, which favoured the lognormal distribution. We changed our analyses accordingly to a linear mixed effects model of logarithmized phase durations, resulting in normality of residuals.

Our second research hypothesis aimed at probing a potential influence of induced affect on dominance ratios. To investigate this, we calculated the proportion of time with which one stimulus variant was reported to be seen relative to the overall time that any of the stimulus variants were reported, per condition (stimulus set and music valence). In contrast to the first research hypothesis, we thus had to aggregate individual responses per condition and we did not include continuous questionnaire data as predictors in the analysis. Accordingly, to evaluate the second hypothesis, we chose a repeated measures ANOVA on dominance ratios, including the predictors stimulus set (Gabors, faces) and music valence (positive, neutral, negative). We checked the normal distribution of values by condition using the Shapiro–Wilk test. If sphericity was observed (as indicated by the Mauchly's sphericity test), we report ɛ and Greenhouse–Geisser corrected p-values.

## Results

**Figure 2:**
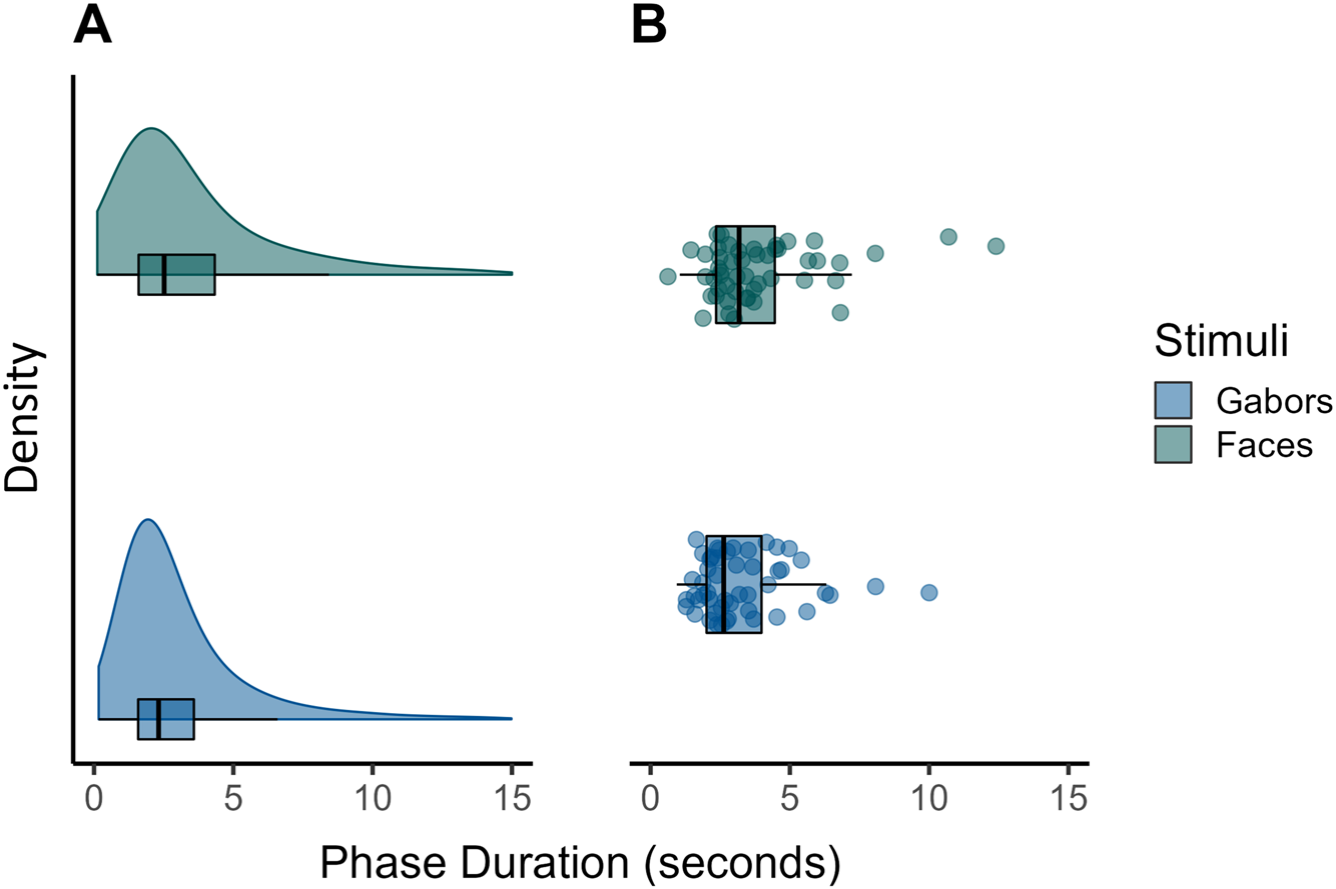
(**A**) Density distributions by stimulus of phase durations (in s) and corresponding boxplots of all given responses (n = 27,145). (**B**) Individual median values of participants by stimulus type and a corresponding boxplot.

Phase durations generally followed a right-skewed distribution with an average median of 3.39 s (Fig. 2). Mixed percepts were generally rare (5.13% of all responses), accounting for 4.38% of all viewing time. The descriptive statistics presented in Table 1 reveal a wide spectrum of affective state and trait scores within the study sample. The observed questionnaire values show high overlap with the ranges that have been observed in the general population^35–37^. The Shapiro–Wilk test confirmed normality of all questionnaire score distributions.

**Table 1:** Descriptive statistics of questionnaire data.

Construct	Questionnaire	*M*	*SD*	Min	Max
Affective traits	PHQ-9	7.38	4.87	0.00	21.00
	STAI-T	39.66	10.21	20.00	66.00
Affective states	PANAS-P	21.34	3.01	14.00	29.00
	PANAS-N	23.76	4.47	12.00	32.00

*PHQ-9* Patient Health Questionnaire-9 measures depressive symptomatology, *STAI-T* State-Trait Anxiety Inventory (trait scale) measures trait anxiety, *PANAS-P/N* Positive and Negative Affect Schedule measure positive and negative affective state.

**Figure 3:**
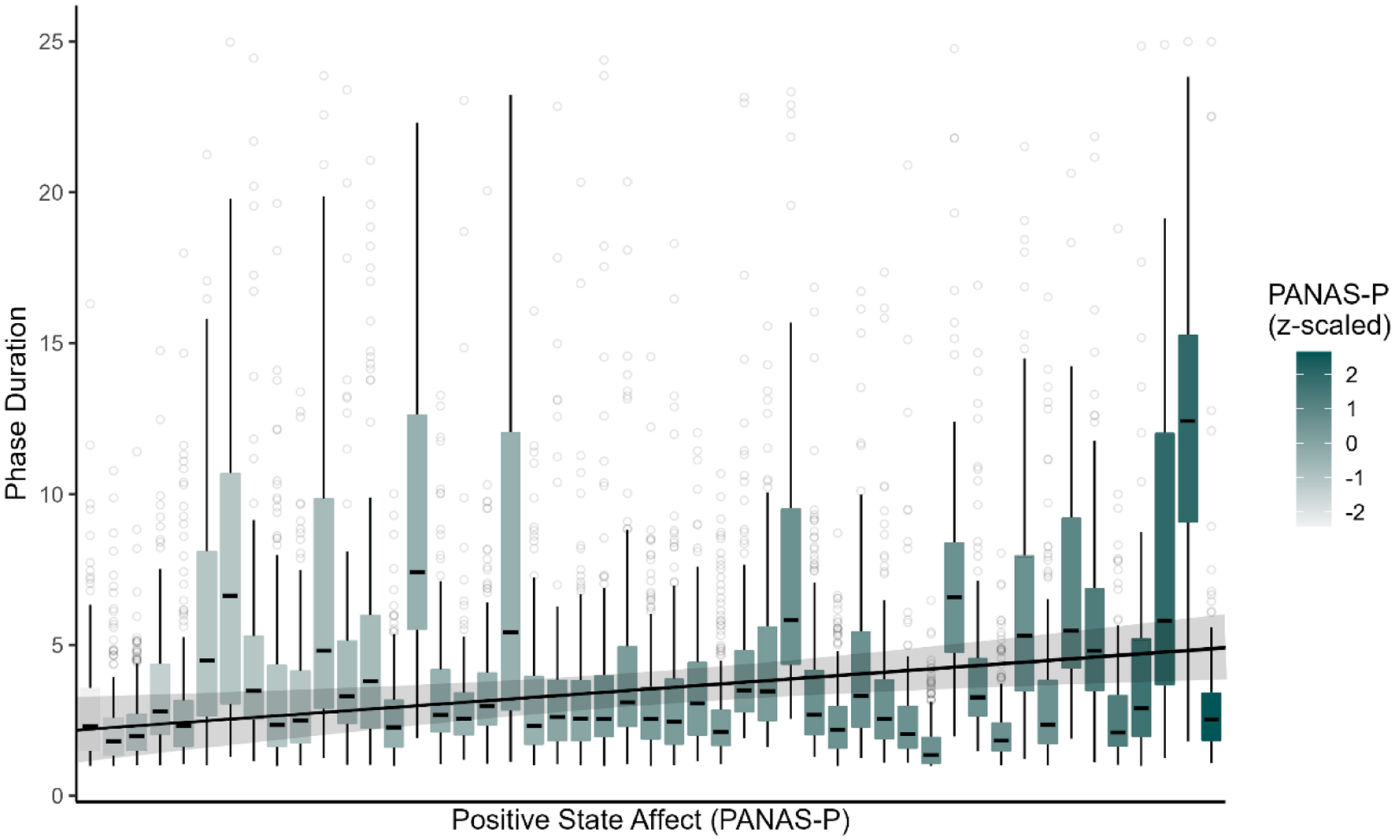
Boxplots of phase durations during neutral trials by participant, ordered along the x-axis by PANAS value (indicated by color gradient). Further depicted is a regression line and 95% confidence interval. Note, that the regression slope is calculated under the assumption of a linear progression of PANAS-P scores from lowest to highest observed value. However, some participants had equal PANAS-P values, which leads to slight deviations between phase duration values predicted by the LMM model and the plotted values of the regression line.

To test for a potential influence of affective states and traits, we calculated a mixed effects regression model in which phase durations of trials with neutral valence were predicted by four questionnaire values (STAI-T, PHQ-9, PANAS-P, PANAS-N, each mean-centered) as well as stimulus type (Gabors, faces). Note, that in the preregistration process, we planned this model to consist of untransformed phase durations, assuming they would follow a gamma distribution (GLMM). However, since regression diagnostics indicated a better fit of the observed durations to a log-normal distribution (see “Methods” section), we changed this analysis to a LMM of log-transformed phase durations. Stimulus type had a significant influence on phase durations (χ^2^ (1) = 11.16, p < 0.001). Specifically, Gabors were associated with lower phase durations than faces (2.68 vs. 3.18 s median duration, see Fig. 2). Contrary to our expectation, affective trait scores for depressiveness or anxiety did not show any significant relationship with phase durations (PHQ-9: χ^2^ (1) = 1.42, p = 0.233; STAI-T: χ^2^ (1) = 0.02, p = 0.889). Baseline positive state affect (as measured by the PANAS-P scale) did show a positive relationship with phase durations (χ^2^ (1) = 6.76, p = 0.009, Fig. 3), suggesting longer (i.e., more stable) percepts when baseline positive affect was higher. For baseline negative affect, the reverse, albeit not significant, trend was observed (PANAS-N: χ^2^ (1) = 3.06, p = 0.080). Importantly, given the nature of both variables, they were highly correlated (r = − 0.700) and should therefore not be considered as independent predictors of phase durations. In order to further explore a potential relationship between state affect and phase durations, we performed an additional, exploratory (i.e., not preregistered) analysis, in which we extended the analysis to the full dataset and added valence of the mood induction condition as a fixed effect predictor. However, music valence did not turn out to be a significant predictor of phase durations (χ^2^ (2) = 2.52, p = 0.284) and did not interact with stimulus type (χ^2^ (2) = 1.38, p = 0.503). This suggests a potential deficit of the mood induction paradigm to influence participants’ affective state in the intended direction.

**Figure 4:**
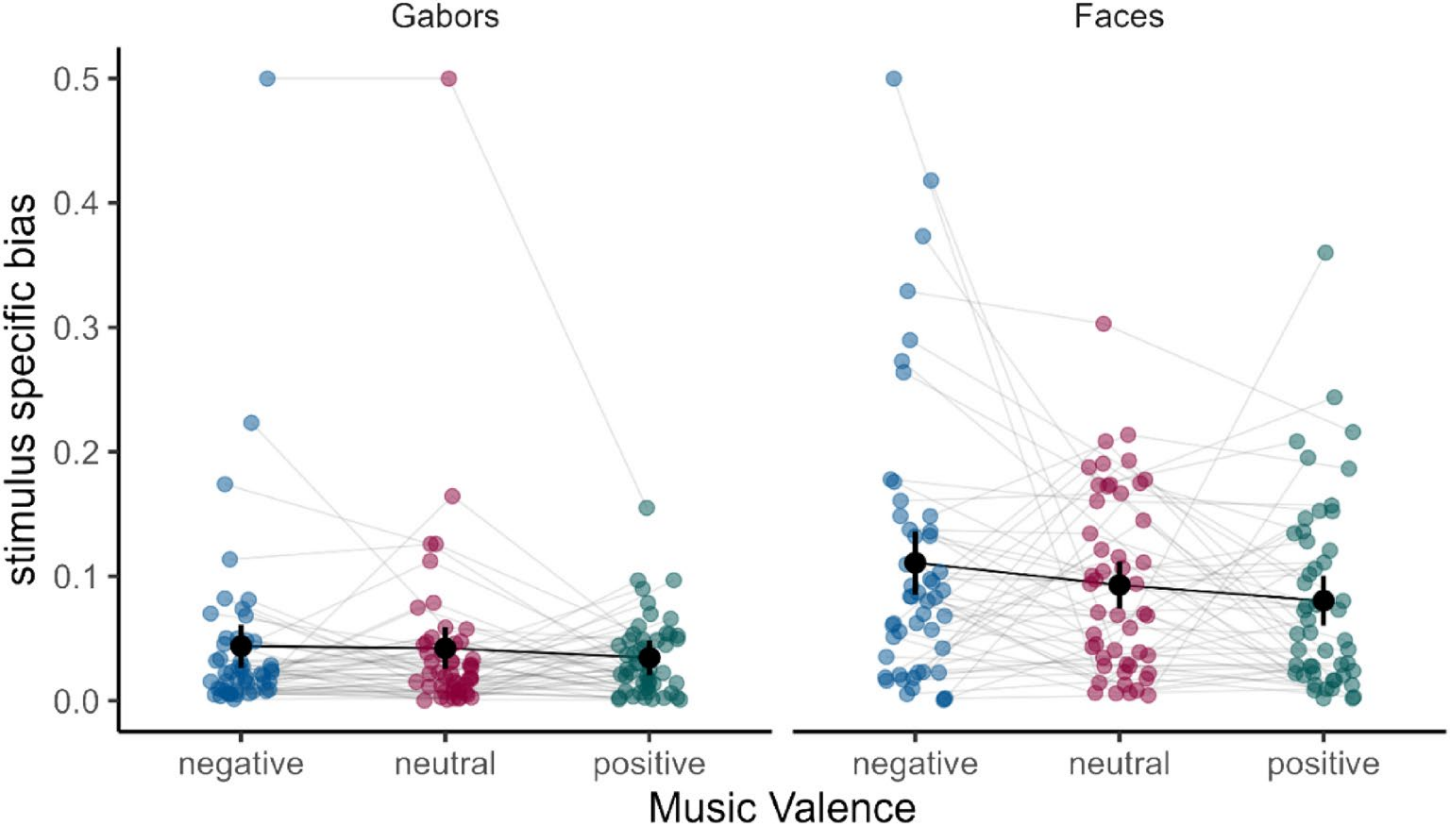
Average bias towards a specific stimulus variant per participant and experimental condition (music valence and stimulus type), as measured by difference from an unbiased (i.e., 50:50) proportion of both stimulus variants. Black bars represent group averages as well as 95% confidence intervals for within subject designs. The face stimulus, compared to the gabors, induces variant specific biases, since upright faces are preferentially perceived when competing with tilted faces. This effect is however not moderated by induced affective valence.

The second preregistered main analysis was aimed at investigating a potential link between music valence and preferential processing of a particular stimulus variant. To do this, we calculated dominance ratios per experimental condition (stimulus type and music valence) and participant. To determine an estimate of individual bias towards a particular stimulus variant, we calculated difference values of dominance durations from 0.5 (i.e., the value to be expected if no bias was present). The resulting bias values were then predicted within a repeated measures ANOVA by stimulus type (Gabors, faces) and musical valence (negative, neutral, positive). As expected, stimulus type had a significant influence on bias values (F(2, 49) = 25.39, *p* < 0.001, η_p_^2^ = 0.11). While there was a significant bias towards a particular stimulus variant in the face condition (upright face reported 56.75% of the time, SD = 8.32), this was not the case for Gabor stimuli (leftward tilted Gabor reported 50.16% of the time, SD = 4.39). However, music valence did not show a significant influence on bias values (F(2, 98) = 2.55, *p* = 0.083, η_p_^2^ = 0.01), and there was no significant interaction effect of music valence and stimulus type (F(2,98) = 0.89, *p* = 0.355, η_p_^2^ = 0.00; ɛ = 0.89; see Fig. 4).

In order to control for potential carryover effects of the mood induction (i.e., negative mood in one block influencing designated neutral mood in the following block), we carried out another exploratory analysis in which we limited the analysed data to only the first experimental block. Note that, since the order of music valence was counterbalanced across participants, it was hence treated as a between-subject predictor in this analysis, which resulted in three independent samples of reduced sizes (neutral, n = 17; negative, n = 17; positive, n = 16). Using this approach, stimulus type, (F(1, 47) = 22.13, *p* < 0.001, η_p_^2^ = 0.20), as well as music valence (F(2, 47) = 5.07, *p* = 0.010, η_p_^2^ = 0.09) yielded significant effects on bias values. Both factors did not significantly interact (F(2, 47) = 2.73, *p* = 0.076, η_p_^2^ = 0.06). Pairwise post-hoc unpaired t-tests revealed that bias of participants in the negative condition was significantly lower than in the neutral (t(31) = − 3.32, *p* = 0.002) and in the positive condition (t(31) = − 2.27, *p* = 0.030). Values for the positive and the neutral mood condition did not differ significantly from one another (t(32) = − 0.99, *p* = 0.331; see Supplementary Fig. S1). This suggests that bias towards a particular stimulus variant was lowest when affective valence was negative.

In a last step, to better understand the relationship between the different measures of perceptual stability in our dataset, we computed bias scores and median phase durations across all stimuli and valence conditions per participant and correlated them. There was a significantly positive correlation (r(48) = 0.54, *p* < 0.001). This correlation remained significant even when the analysis was conducted separately for both stimulus types, which challenges the assumption of phase durations and dominance ratios being largely independent measures of perceptual stability during binocular rivalry.

## Discussion

The aim of this study was to further examine the potential relationship between affective states and traits to different measures of perceptual stability during binocular rivalry, given that previous research has yielded an overall inconclusive pattern of results. Here, we found that the two analysed measures of perceptual stability followed a pattern to be expected in a binocular rivalry task in healthy participants. Phase durations were generally right skewed, with most values falling into a range between 2 and 10 s. We also found that stimulus type had a significant influence on phase durations, i.e., differently oriented Gabors tended to alternate faster than differently oriented faces. This effect may be caused by differences in low-level features or differences in familiarity between the stimuli, as both have been shown to affect phase durations^38,39^. We also found that stimulus type influenced dominance ratios, i.e., stimulus orientation affected the likelihood of a particular stimulus variant being perceived significantly more in faces than in Gabors, as was intended to quantify stimulus-specific bias.

Importantly, the two measures of perceptual stability in our study, could, in principle, be independent from each other (i.e., one could hold the amount of perceptual alternations in a given time constant but change the ratio with which both variants are perceived and vice versa). While many studies^12,16,22^, including ours, rest on this assumption of independence and treat both measures as outcome variables in separate statistical analyses, our results emphasize their interdependent nature. Hence, in a critical evaluation of the presented results, we suggest categorizing them as different measures of perceptual stability during binocular rivalry.

Contrary to our hypotheses as well as previously described findings, affective traits did not significantly predict variance in phase durations. Importantly, the design of this study differed from previous ones in two ways. Firstly, previous studies have mostly focused on affective stimuli, e.g., bistable point walkers that could be perceived as either walking towards or away from the observer^40^. The results could therefore stem from well-established differences in attention allocation, i.e., more socially anxious people will draw more attention towards the more anxiety provoking stimulus variant (e.g., a walker that is approaching the participant). Since our stimuli were deliberately chosen to be comparatively affectively neutral, such attentional biases will not have had a substantial impact on alternation rates. Secondly, studies that have shown alternation rates to be elevated even when stimulus valence was neutral all showed this effect in clinical populations^14–16,41^. Since in all the studied disorders (generalized anxiety disorder (GAD), major depression, bipolar disorder, schizophrenia) psychomotor, as well as cognitive functions, have been shown to be impaired^42,43^, this is an important potential confound to be considered. However, Jia et al.^14,15^ have included catch trial conditions (trials in which both eyes receive the same input and perceptual alternations are thus the veridical percept), to control for potential behavioural differences of reporting current percepts and have not found significant differences between the groups. Furthermore, other authors^16,41^ have included different tests of psychomotor functions and cognitive processing in order to rule out that differences in these constructs play a moderating role in explaining group differences in perceptual alternations. Nevertheless, it is conceivable that potentially important variables in which the diagnostic groups differed (e.g., susceptibility to mixed percepts or tendency to report them), were not considered as possible confounds in the experimental designs.

Notably, Jia et al.^15^ have found diverging directions of influence of anxiety and depression, i.e., depression was correlated to fewer perceptual alternations, whereas anxiety was correlated to more. Such effects are unlikely to occur in the general population, as trait anxiety and depressiveness usually are highly correlated, which in the case of our study is reflected by the multicollinearity of the regression model. For the purpose of their study^15^, the authors differentiated between GAD and depression patients, two disorders of which some comorbidity estimates reach up to 70%^44^. It thus seems unlikely that the effects described in their study are attributable to separate and opposing effects of different affective traits on perceptual stability. Rather, they might stem from a highly selective sampling process in which two characteristics that are usually highly interdependent were separated. Thus, several of the previously reported effects of affective traits on phase durations are best explained by attentional mood congruency biases of participants and selection biases concerning the participant sample. So far, only one study has found a statistically significant (positive) correlation between alternation rates of neutral stimuli and affective traits in a sample of healthy participants^22^. However, the effect sizes were comparably small and findings were inconsistent across different stimulus categories. The results presented in this study do not support a strong link between affective traits and alternation rates in the general population, but further research is needed to investigate a potential role that low and high-level stimulus features could play in determining the link between observer based variables such as affective traits and phase durations during binocular rivalry.

We further analysed affective state and its relationship with predominance ratios. In our preregistered main analysis, we did not find a significant effect of one's affective status on preferential processing of a particular stimulus variant, adding to a generally inconsistent pattern of results. There are two previous studies that support this potential influence during perceptual rivalry with moderate to strong effect size estimates. Pettigrew and Carter^21^ found a positive relationship between positive state affect and the proportion of time that dots were perceived compared to not perceived during motion induced blindness (MIB). Although the authors conceive MIB as a form of perceptual rivalry and both phenomena resemble each other in their temporal alternation dynamics, it has been disputed whether their appearance underlies a common neural mechanism ^45^. Sheppard and Pettigrew^20^ showed a strong influence of positive affect measured at the beginning of the experiment on the predominance proportion of the two potential stimulus interpretations during plaid motion rivalry (PMR). They further showed strong correlations (r = 0.84, *p* < 0.001) of alternation rates during binocular rivalry and PMR, suggesting an underlying common mechanism contributing to both phenomena. However, in contrast to our study, both studies focused on baseline affective state without manipulation of participants' mood.

In our dataset, baseline affective state did show a significant positive relationship with phase durations, suggesting an enhancing effect of positive affect on perceptual stability. Importantly, we have manipulated participants affective state and did not correct for potential carryover effects. To rule out such carryover effects, we carried out an additional analysis which only included the first valence block. Notably, this analysis was of an exploratory nature and was carried out in a limited subset of the data, likely resulting in reduced statistical power. However, the results of this analysis found negative affect leading to less bias towards a particular stimulus variant. Taken together, both analyses indicate that affective states affect perceptual stability during binocular rivalry with respect to their valence. While we found higher baseline positive affect to be associated with longer phase durations, induced negative affective valence was related to attenuated bias towards a particular stimulus variant. This relationship is congruent with our preregistered hypotheses and with recent research from our group which demonstrated reliance on priors during a task of perceptual filling-in to be associated with induced affective valence^23^.

The fact that an effect of the mood condition only was significant in data of the first experimental block could indicate that the mood induction paradigm did not work as intended. As there was no significant difference between the neutral and the positive condition with respect to their influence on dominance ratios, the positive music condition might have failed in elevating participants mood above their baseline values. Diener et al.^46^ found that, overall, the effect of mood induction procedures is comparatively low. They further found that while, for instance, a negative mood induction often leads to more negative mood ratings than a positive mood induction, overall values of affective valence are still on the positive side of the two-sided valence continuum. It is further conceivable that other factors that influenced participants’ mood (e.g., exhaustion or fatigue) gained in relative influence, compared with the mood induction, over the time course of the experiment. Future experiments should therefore control for the effectiveness and validity of the mood induction procedure through repeated mood measurements, pauses between experimental blocks and between-subject designs in large samples. Furthermore, variations in baseline mood across participants provide a valuable tool to explain variance in perceptual stability, as they are less prone to the described methodological confounds.

However, the presented findings are in line with an affective realism approach to the debate over cognitive penetrability of perception, in which the affective state of an observer is taken into account in the perception of its environment^9^. This is especially noteworthy, since in the outlined experimental design all stimuli were affectively neutral and the results stem from a sample of the general population. Neither attention mediated mood congruency effects nor selective sampling and confounding characteristics of clinical populations are thus likely explanations of the described findings. Nevertheless, given their inconsistent nature (e.g., only negative but not positive induced mood leading to changes in dominance ratios), these results are to be interpreted with caution.

## Supplementary Information

Supplementary Figure S1.

## Data Availability

All collected data, visual stimuli as well as the analysis script are publicly available under https://osf.io/2gyvu/.
