# Supplementary material for: The impact of affective states and traits on perceptual stability during binocular rivalry
Source: Sci Rep. 2023 May 17;13:8046. doi: 10.1038/s41598-023-35089-5 (PMC10192310; doi:10.1038/s41598-023-35089-5)
Supplement: Supplementary file 1 — Supplementary Figure S1. [file 41598_2023_35089_MOESM1_ESM.docx]

**Supplementary Material**


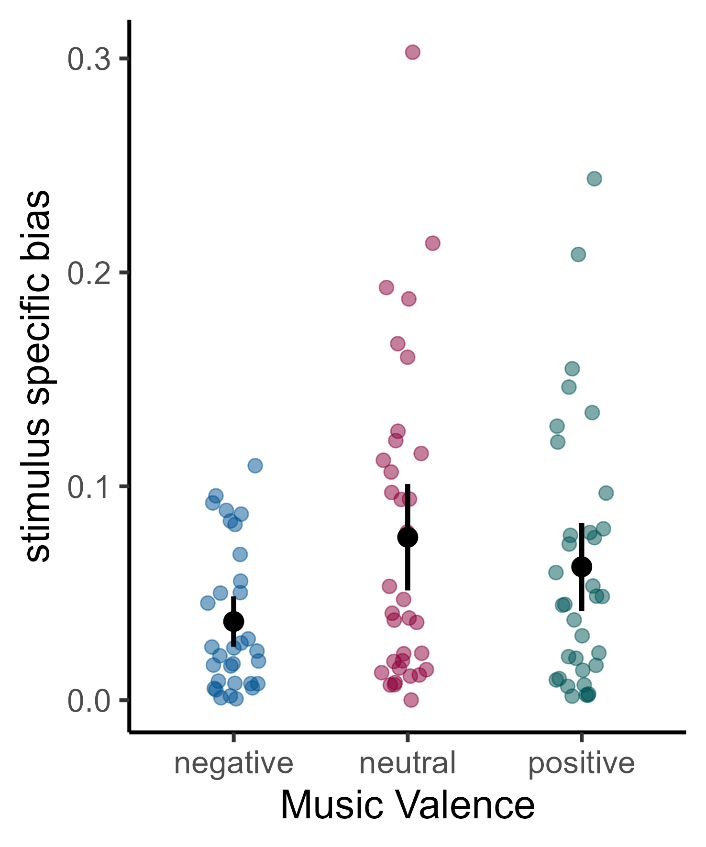


**Figure S1** Average bias scores (i.e., deviance of proportion with which both stimulus variants were reported from .5) an 95% confidence interval by participant and condition in only the first experimental block. Note, that since order of music valence was counterbalanced, the three conditions include three independent samples (N’s= 16, 17 and 17).
